# ANALYSIS OF REPORTING GUIDELINE AND TRIAL REGISTRATION POLICIES AMONG GERIATRIC JOURNALS: A CROSS-SECTIONAL STUDY

**DOI:** 10.1093/geroni/igad104.2331

**Published:** 2023-12-21

**Authors:** Shaelyn Ward, Logan Corwin, Jacob Duncan, Caleb Smith, Danya Brewer, Griffin Hughes, Matt Vassar

**Affiliations:** Oklahoma State College of Osteopathic Medicine at the Cherokee Nation, Tahlequah, Oklahoma, United States; Oklahoma Center for Health Sciences, Tulsa, Oklahoma, United States; Oklahoma State College of Osteopathic Medicine at the Cherokee Nation, Tulsa, Oklahoma, United States; Oklahoma State College of Osteopathic Medicine at the Cherokee Nation, Tulsa, Oklahoma, United States; Oklahoma State College of Osteopathic Medicine at the Cherokee Nation, Tulsa, Oklahoma, United States; Oklahoma Center for Health Sciences, Tulsa, Oklahoma, United States; Oklahoma Center for Health Sciences, Tulsa, Oklahoma, United States

## Abstract

The use of reporting guidelines and clinical trial registration policies by academic journals reduces bias and improves transparency in clinical research. It is unknown whether geriatric and gerontology journals mention, recommend, or require their use for the studies they may potentially publish. The purpose of this study is to assess the submission guidelines of the top geriatric and gerontology journals for their editorial recommendation or requirement of predetermined reporting guidelines and clinical trial registration. We identified the top 100 journals in the “Geriatrics and Gerontology” subcategory using the 2021 Scopus CiteScore tool. We probed each journal’s “Instructions to Authors” for statements regarding reporting guidelines for popular study designs and extracted them as “Not Mentioned”, “Recommended”, “Does Not Require”, or “Required”. Further, we classified each journal’s reference to clinical trial registration in a similar manner. Of the 100 examined journals included in our analysis, the QUOROM statement was not mentioned by any journals, whereas the CONSORT statement (44/100, 44%) was mentioned and recommended/required the most often. PRISMA guidelines were not mentioned by 57 journals (57/100,57%). Furthermore, 45 journals (45/100,45%) did not mention study registration. Therefore, the recommendation or requirement of reporting guidelines and clinical trial registration in the top 100 geriatric and gerontology journals is inconsistent. Journal editors should strongly recommend that author’s follow validated reporting guidelines to reduce potential bias and improve transparency in the articles they publish.

